# Dual experience replay enhanced deep deterministic policy gradient for efficient continuous data sampling

**DOI:** 10.1371/journal.pone.0334411

**Published:** 2025-11-11

**Authors:** Teh Noranis Mohd Aris, Ningning Chen, Norwati Mustapha, Maslina Zolkepli

**Affiliations:** Department of Computer Science, Universiti Putra Malaysia, UPM, Serdang, Selangor, Malaysia; University of Lagos Faculty of Engineering, NIGERIA

## Abstract

To address the inefficiencies in sample utilization and policy instability in asynchronous distributed reinforcement learning, we propose TPDEB—a dual experience replay framework that integrates prioritized sampling and temporal diversity. While recent distributed RL systems have scaled well, they often suffer from instability and inefficient sampling under network-induced delays and stale policy updates—highlighting a gap in robust learning under asynchronous conditions. TPDEB significantly improves convergence speed and robustness by coordinating dual-buffer updates across distributed agents, offering a scalable solution to real-world continuous control tasks. TPDEB addresses these limitations through two key mechanisms: a trajectory-level prioritized replay buffer that captures temporally coherent high-value experiences, and KL-regularized learning that constrains policy drift across actors. Unlike prior approaches relying on a single experience buffer, TPDEB employs a dual-buffer strategy that combines standard and prioritized replay Buffers. This enables better trade-offs between unbiased sampling and value-driven prioritization, improving learning robustness under asynchronous actor updates. Moreover, TPDEB collects more diverse and redundant experience by scaling parallel actor replicas. Empirical evaluations on MuJoCo continuous control benchmarks demonstrate that TPDEB outperforms baseline distributed algorithms in both convergence speed and final performance, especially under constrained actor–learner bandwidth. Ablation studies validate the contribution of each component, showing that trajectory-level prioritization captures high-quality samples more effectively than step-wise methods, and KL-regularization enhances stability across asynchronous updates. These findings support TPDEB as a practical and scalable solution for distributed reinforcement learning systems.

## 1. Introduction

Reinforcement learning learns through trial and error and typically relies on a large amount of experience data from interactions with the environment, which results in inefficient data collection and agent training. Training reinforcement learning agents requires massive amounts of interaction data. For example, training a DOTA 2 agent to reach human-level performance requires the equivalent of 45,000 years of game expe-rience [1], while training a top-tier StarCraft II agent requires 10 million match samples [2]. In contrast, human players can master the same skills without requiring such ex-tensive time or an equivalent amount of data. To this end, researchers have attempted breakthroughs along two fronts: accelerating sample collection and improving the learning efficiency of experiences through efficient learning algorithms. Policy gradient algorithms [3–5], due to their conceptual simplicity and strong generalizability, have become mainstream reinforcement learning methods. This paper, based on policy gra-dient algorithms, analyzes the bottlenecks in data collection and sample learning effi-ciency in reinforcement learning. A straightforward approach to improving data col-lection efficiency is parallelization. Algorithms such as A3C [6] and Gorila [7] deploy multiple agents to interact in parallel, synchronously collecting samples in independent environments. In a parallel framework, the module that aggregates policy replica sam-ples/gradients from threads is known as the learner, while the threads that interact with the environment are called workers. In A3C, workers periodically upload accumulated gradients to the learner’s policy network and synchronously update their local policies. In A2C [8], workers conduct local interactions, with the learner centrally generating and distributing actions, and samples are eventually returned for batch gradient training. This model suffers from a synchronization bottleneck: if one worker takes too long to collect data, the samples from other workers must wait for synchronization, limiting efficiency to that of the slowest node. Cong Lu et al. [9] addressed the issue of data scarcity by proposing a synthetic experience replay mechanism (SynthER), which em-ploys generative modeling for flexible sampling and validates its effectiveness in both cross-modal (proprioceptive/pixel) and training modes (offline/online). Shi Shengmiao et al. [10] proposed categorized experience replay, where DDPG samples are classified using a dual criterion of TD error and immediate rewards, and a dual replay Buffer is constructed for prioritized storage; experiments demonstrate that this method signifi-cantly improves performance. IMPALA [11] decouples the learner and workers, with workers continuously collecting samples and storing them in a replay Buffer, thereby enabling continuous data collection. However, this method suffers from a policy lag issue: worker policies are not updated synchronously with the learner, leading to dis-tributional shifts in data and unstable gradients. Longfei Zhang et al. [12] proposed a three-module experience replay architecture (dual replay bodies, prioritized replay body, and normal replay body), which supplies samples differentially according to the training stage; D4RL benchmark tests indicate that it surpasses the current state-of-the-art offline algorithms. Enhancing data collection and sample utilization efficiency is central to accelerating the skill acquisition of reinforcement learning agents. Distributed reinforcement learning (DRL) has emerged as a promising paradigm for scaling learning across multiple agents. However, asynchronous sampling and decentralized experience sharing introduce challenges such as inefficient buffer utilization, reduced sample diversity, and unstable policy convergence. These issues are particularly pronounced in continuous control tasks, where timely and diverse experience replay is critical. Recent advances by Shirmohammadi et al. have significantly contributed to energy-efficient communication and hardware-level optimization in intelligent systems. JoBiS [13] introduces a joint capacitance and inductance bit stuffing algorithm for multi-chip deep learning accelerators, achieving remarkable reductions in power consumption and delay. Similarly, LPRLC [14] proposes a predictive coding scheme to minimize energy usage in wireless body area networks, while their work on EH-WSNs [15] leverages deep reinforcement learning(Q- learning) to enhance throughput under energy constraints. Additionally, approximate algorithms for WSN coverage [16] employed bipartite graph models to optimize sensor deployment to optimize sensor deployment and extend network lifetime. These contributions reflect deep expertise in energy-aware system design, coding efficiency, and adaptive learning mechanisms. Notably, they also proposed a dynamic sampling method based on change rate (DSCR) [17] for energy-harvesting body sensor nodes, which intelligently adjusts sampling rates according to real-time energy availability. Their work introduces an adaptive energy manager that classifies sensors into three energy-level tiers, enabling differentiated sampling policies that significantly reduce unnecessary data acquisition. This contribution represents a highly practical and elegant solution to the challenge of energy neutrality in Wireless Body Area Networks (WBANs), and has set a benchmark for sustainable sensing in constrained environments. They primarily focus on hardware-level transmission optimization, energy harvesting, and single-agent scheduling strategies. In contrast, our work addresses a complementary challenge: improving sample efficiency and policy stability in asynchronous distributed reinforcement learning environments.

Despite the growing interest in distributed reinforcement learning, existing sampling strategies often suffer from inefficient experience utilization and unstable policy updates, especially under asynchronous conditions. These limitations hinder scalability and convergence in real-world applications. By situating TPDEB within the broader landscape of reinforcement learning and energy-aware optimization, we aim to complement and extend the foundational work of Shirmohammadi et al., offering a scalable solution for high-throughput, distributed learning systems.

On the other hand, optimizing sample utilization efficiency is also a key challenge in reinforcement learning. Neuroscientific studies have shown that the hippocampi of rodents frequently replay experience sequences during both wakefulness and sleep, and the replay frequency for high-reward experiences is significantly increased [18–20]. In reinforcement learning, the temporal difference (TD) error is the core metric for evalu-ating samples. In curiosity-driven exploration methods, TD error guides the choice of exploration direction [21]. Samples with high TD errors are typically prioritized for re-use [22]. Research [23,24] proposed a prioritized experience replay (PER) strategy that dynamically assigns sampling weights based on the TD error. This method differentiates the learning frequencies of samples, significantly enhancing the utilization of critical data. Dan Horgan et al. [25] proposed a distributed prioritized experience replay framework that decouples action generation from learning: actors interact with the environment using a shared network and store samples in a shared memory Buffer, while learners replay these samples to update the network, thereby significantly improving algorithm efficiency. Building on PER, Baturay Saglam et al. [26] designed a novel sampling framework, Actor-PER, for Actor-Critic methods, which integrates stability optimization and an analysis of PER’s shortcomings; Experiments have demonstrated that its performance exceeds that of baseline algorithms. Botvinick, M. et al. [27] Point out that the fundamental challenge in reinforcement learning lies in its sample inefficiency-requiring extensive data samples to achieve effective learning. Their approach systematically addresses the dual root causes of this limitation: Episodic deep RL resolves the issue of incremental parameter adjustment, episodic methods focus on sparse memory retrieval or curriculum-driven replay. Peiquan Sun et al. [28] Point out Stale states (generated by outdated policies) are ineffective for updating the current policy. Consequently, it is desirable to compare the state distribution in the replay buffer with the current state distribution and prioritize replaying transitions with higher similarity to enhance convergence performance. Marc Brittain et al. [29] propose a novel experience replay mechanism to enhance training efficiency and performance. This approach assigns decayed priorities to transitions preceding the currently sampled transition, and incorporates a technique to fine-tune the decay rate. Method [30] performs optimization using the same set of transitions (sampled as part of a mini-batch) this approach addresses The actor’s inability to learn effectively from transitions with high TD-error. and The deviation of the approximated policy gradient (under the Q-network) from the true gradient under the optimal Q-function. Some off-policy algorithms, such as DQN or DDPG, achieve high sample efficiency by repeatedly performing random sampling on interaction data from the experience replay buffer for learning. However, the practice of uniform random sampling from the experience replay buffer overlooks the varying importance of different samples for training the agent. To address this issue, Zhuoying Chen et al. [31,32] effectively resolved this problem by proposing ALAP [31] and DALAP [32] have explored adaptive importance-sampling correction techniques to enhance learning efficiency in off-policy settings. ALAP introduces a self-attention network to dynamically tune the correction coefficient β during training, to adaptively adjust importance sampling weights. DALAP builds on this by employing a parallel attention mechanism to estimate distributional shift and by proposing a neighbor-based priority augmentation scheme, to quantify distributional shift and enrich experience diversity through a neighbor-prioritization strategy. These contributions provide valuable insights into mitigating the limitations of standard PER and improving the robustness of experience sampling.

Building on these advances, we propose the Trajectory-level Prioritized Dual Experience Buffer (TPDEB), which offers a complementary direction: enhancing sample efficiency and policy stability in distributed, asynchronous settings. TPDEB departs from single-agent correction frameworks and instead emphasizes scalable architecture via parallel policy replicas, trajectory-based experience ranking, and a KL-regularized actor update. In doing so, our work aligns with the same goals of improving learning robustness, while addressing the additional challenges posed by multi-agent and high-throughput environments. Compared to prior methods, TPDEB offers a more scalable and adaptive solution for asynchronous DRL environments. Our contributions are threefold: 1) Introduce a dual-buffer mechanism tailored for asynchronous environments: We propose a trajectory-level prioritized dual replay buffer, where full trajectory sequences are ranked and sampled based on cumulative TD-error to improve sample quality and coherence. 2) Demonstrate its effectiveness through extensive experiments on continuous control tasks: We develop a distributed sampling architecture with multiple asynchronous policy replicas, increasing throughput and state-action diversity. 3) Provide theoretical insights into its convergence behavior: We incorporate a KL-divergence penalty between replicas into the actor’s update rule, helping maintain policy alignment and improve training stability. These advances position TPDEB as a scalable and scientifically grounded solution for distributed reinforcement learning.

## 2. Materials and methods

### 2.1. Parallel sample collection based on policy replicas

#### 2.1.1. Accelerating sample collection with policy replicas.

**Fig 1:**
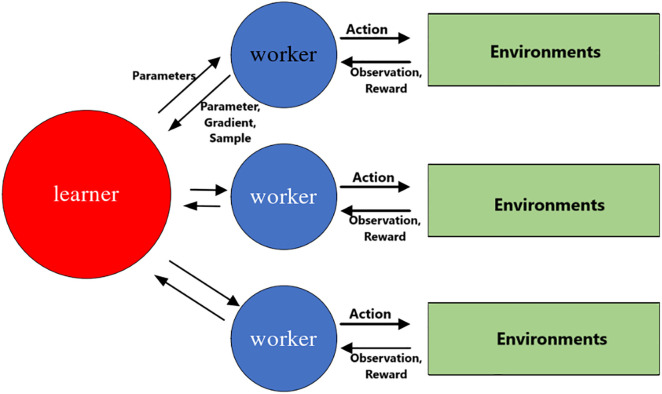
Parallel experience collection structure.

The basic structure for parallel experience collection using policy replicas is shown in Fig 1.

Each worker synchronizes the parameters of the learner’s policy and value networks and collects experience data in parallel within independent environments. Workers transmit the samples to the learner, which performs centralized training. In another mode, workers compute gradients independently and submit them to the learner to update the learner’s network parameters.

Policy gradient algorithms are a class of on-policy reinforcement learning methods, meaning that the experience data is discarded after being used for an update. In contrast, off-policy reinforcement learning algorithms utilize an experience replay Buffer, allowing the experience data to be reused. To improve sample utilization, policy gradient algorithms also repeatedly learn from the existing experience data. However, when the updated policy network processes old data, the distributions of states and actions no longer match. From another perspective, repeatedly learning from old data is equivalent to the agent learning from experiences collected by other agents.

Let $\pi_{\theta }$ represent the policy function of the current learning agent, and $\pi_{\varphi }$ represent the policy function replica used for experience collection, where θ and φ are the neural network parameters of the two functions, respectively. Represent the steady-state distribution of states as:


$$\rho_{\pi }(s)=\int_{S}\sum {\mathrm{\backslash nolimits}}_{t=\text{1}}^{\infty }\gamma^{t-\text{1}}p(x\to s,t,\pi )dx$$ (1)

p(x→s,t,π) represents the probability of reaching state s from state x after t steps following policy π.

When a worker collects experience using its own policy, the state distribution is $\rho_{\varphi }(s)$, while the objective function for the learner utilizing the experiences collected by policy φ is:


$$J_{\varphi }\left(\pi_{\theta }\right)=\int_{S}\int_{A}\rho_{\varphi }(s)\pi_{\theta }(s,a)Q(s,a)dads$$ (2)

The gradient with respect to parameter θ is:


$$\nabla_{\theta }J_{\varphi }\left(\pi_{\theta }\right)\approx \int_{S}\int_{A}\rho_{\varphi }(s){\nabla_{\theta }\pi }_{\theta }(s,a)Q(s,a)dads$$ (3)

Using policy gradients with baseline, the result is:


$$\begin{array}{cc} \nabla_{\theta }J_{\varphi }\left(\pi_{\theta }\right)\approx \int_{S}\int_{A}\rho_{\varphi }(s){\nabla_{\theta }\pi }_{\theta }(s,a)A(s,a)dads & =\int_{S}\rho_{\varphi }\int_{A}\pi_{\varphi }\cdot \frac{\pi_{\theta }}{\pi_{\varphi }}\nabla_{\theta }\text{log}\pi_{\theta }(s,a)A(s,a)dads\\ & =E_{s\sim \rho_{\varphi },a\sim \pi_{\varphi }}\left[\frac{\pi_{\theta }}{\pi_{\varphi }}\nabla_{\theta }\text{log}\pi_{\theta }(s,a)A(s,a)\right] \end{array}$$ (4)

A(s,a)=Q(s,a)−V(s,a) is called the advantage function.

Equation (4) shows that although data is collected using policy $\pi_{\varphi }$, the gradient is corrected by introducing the importance sampling factor $\frac{\pi_{\varphi }}{\pi_{\varphi }}$ during gradient computation, ensuring that actions are still generated according to policy $\pi_{\theta }$.

When updating parameters according to Equation (4), the algorithm becomes unstable if the importance sampling factor $\frac{\pi_{\theta }}{\pi_{\varphi }}$ is excessively large or small.

The purpose of introducing the importance sampling factor $\frac{\pi_{\theta }}{\pi_{\varphi }}$ is to adjust the coefficient in front of the advantage function to stabilize the gradient. This functionality can be achieved by modifying $\nabla_{\theta }\text{log}\pi_{\theta }(s,a)$ Since $\nabla_{\theta }\text{log}\pi_{\theta }(s,a)=\nabla_{\theta }\pi_{\theta }(s,a)/\pi_{\theta }(s,a)$, the objective function can be re-expressed as:


$$\nabla_{\theta }J_{\varphi }\left(\pi_{\theta }\right)=E_{s\sim \rho_{\varphi },a\sim \pi_{\varphi }}\left[\frac{\nabla_{\theta }\pi_{\theta }(s,a)}{\pi_{\varphi }(s,a)}\hat{A}(s,a)\right]$$ (5)

Based on this gradient, a new objective function is constructed as a replacement.


$${L_{surr}(\pi_{\theta })=E}_{s\sim \rho_{\varphi },a\sim \pi_{\varphi }}\left[\frac{\pi_{\theta }(s,a)}{\pi_{\varphi }(s,a)}\hat{A}(s,a)\right]$$ (6)

$\hat{A}=R(s,a)+\gamma V\left(s^{\prime }\right)-V(s)$ is the estimated value of the advantage function A(s,a). For a batch of collected interaction samples (assuming there are N samples), the batch is used for multiple parameter updates, with each update clipping the update magnitude.


$${L_{surr}(\theta )=E}_{t}\left[\text{min}\left(R_{t}(\theta ){\hat{A}}_{t},clip(R_{t}(\theta ),\text{1}-\in ,\text{1}+\in ){\hat{A}}_{t}\right)\right]=\frac{\text{1}}{N}\sum {\mathrm{\backslash nolimits}}_{t=\text{1}}^{N}\text{min}\left(R_{t}(\theta ){\hat{A}}_{t},clip(R_{t}(\theta ),\text{1}-\in ,\text{1}+\in ){\hat{A}}_{t}\right)$$ (7)

$R_{t}(\theta )=\pi_{\theta }(s,a)/\pi_{\varphi }(s,a)$ is the importance sampling factor. Equation (7) constrains $R_{t}(\theta )$ within the range [1−∈,1+∈], where 0<∈<1.

#### 2.1.2. Soft update of the target policy.

**Fig 2:**
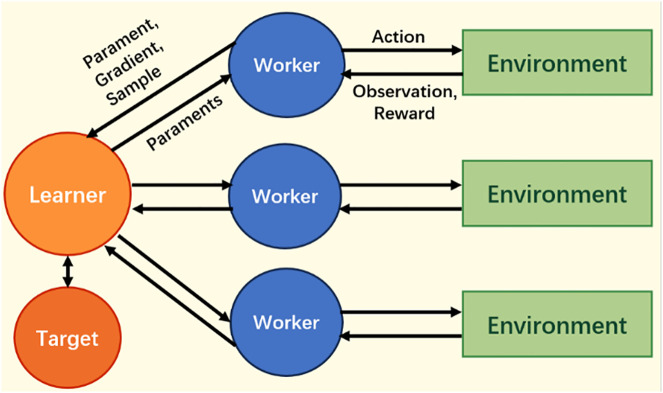
Parallel experience collection with targeted policy networks.


To further stabilize the training process, a target policy is introduced, as shown in Fig 2. The target policy network is a periodic backup of the learner’s policy network [33,34]. Let $\pi_{{worker}_{i}}$ represent the policy network of the i-th replica agent. When calculating the importance sampling factor, the target policy network $\pi_{target}$ is used for correction:

$$R_{t}(\theta )=\frac{\pi_{\theta }(s_{t},a_{t})}{\text{max}\left(\pi_{target}\left(s_{t},a_{t}\right),\beta \pi_{{worker}_{i}}(s_{t},a_{t})\right)}$$ (8)

β(0<β≤1) is used to adjust the constraint range, ensuring that the adjusted importance sampling factor does not exceed $\frac{\pi_{\theta }}{\pi_{target}}$. Additionally, a smaller β will encourage the replica agent to adopt more aggressive strategies for exploration, at the cost of reduced stability. The corrected objective function is:


$$L_{\pi }(\theta )=E_{s_{t},a_{t}}\left[\frac{\pi_{\theta }\left(s_{t},a_{t}\right)}{\text{max}\left(\pi_{target}\left(s_{t},a_{t}\right),\beta \pi_{{worker}_{i}}\left(s_{t},a_{t}\right)\right)}\hat{A}(s_{t},a_{t})\right]$$ (9)

Directly copying the learner’s policy network to the target in the learner and target policy networks can lead to instability in the training process. Let $\theta^{\prime }$ represent the parameters of the target policy network, and its update method is:


$$\theta^{\prime }=\varsigma \theta +(\text{1}-\varsigma )\theta^{\prime }$$ (10)

0<ς≤1 represents the proportion of updates that the target network receives from the learner in each training iteration. This may somewhat slow down the learning process, but the benefits outweigh the drawbacks in terms of stabilizing the training process.

### 2.2. Trajectory-based dual experience replay buffer policy gradient algorithm

#### 2.2.1. Dual experience replay buffer.

Algorithm 1. Dual Experience Replay Buffer

Inputs: maximum capacity of weighted replay Buffer $M_{R}$, maximum capacity of uniform replay Buffer $M_{O}$, number of policy replicas W, initial parameters of policy replica network φ.

1: Initialisation:

2: Initialise the policy network parameters of W workers as φ.

3: Start a thread loop execution for each worker:

4: Collect interaction trajectories τ using the current policy, compute Δ and ${\overset{―}{G}}_{t}$

5: If $(\Delta >\text{min}\;B_{R}:\;$

6: Update $B_{R}$ with τ

7: Update sampling weight $P(i)=I(i)/\sum {\mathrm{\backslash nolimits}}_{k=\text{1}}^{\left|B_{R}\right|}I(k)$ for each sample.

8: Else

9: Update $B_{O}$ with τ

10: until the end of training

Algorithm 1 describes the construction process of the dual experience replay Buffer.

Some off-policy algorithms (such as DQN or DDPG) improve sample utilization by repeatedly and randomly sampling interaction data from the experience replay Buffer for learning. However, uniformly random sampling from the experience replay Buffer overlooks the varying levels of importance that different samples have for agent training. Therefore, the PER algorithm [35] assigns different sampling weights to samples based on TD error, giving samples with larger TD errors a higher probability of being sampled. Building on this, we consider samples as part of trajectories for an overall evaluation. A $\left\langle s_{t},a_{t},r_{t+\text{1}},s_{t+\text{1}}\right\rangle$ is defined as one sample, and its corresponding trajectory $\tau :S_{\text{0}},A_{\text{0}},R_{\text{1}},S_{\text{1}},\cdots S_{T-\text{1}},A_{T-\text{1}},R_{T},S_{T}$ comprises multiple samples. The TD error of a sample is defined as $\delta_{T}=r_{t+\text{1}}+\gamma V\left(s_{t+\text{1}}\right)-V(s_{t})$, and the cumulative TD error of a trajectory is:


$$\Delta_{\tau }=\sum {\mathrm{\backslash nolimits}}_{t-\text{1}}^{T}\left|\delta_{t}\right|$$ (11)

After sorting the trajectories according to $\Delta_{\tau }$, the K trajectories with the largest cumulative error are stored in the prioritized replay Buffer $B_{R}$. Combined with future returns:


$${\overset{―}{G}}_{t}=\frac{\text{1}}{T-t}\sum {\mathrm{\backslash nolimits}}_{k=t}^{T-\text{1}}\gamma^{k-t}r_{t+\text{1}}$$ (12)

Based on $\Delta_{\tau }$ and ${\overset{―}{G}}_{t}$, the sample importance is defined as:


$$I_{t}=\frac{\left|\delta_{t}\right|}{\Delta_{\tau }}{\overset{―}{G}}_{t}$$ (13)

Subsequently, the sampling weights of the samples in the prioritized replay Buffer $B_{R}$ are normalized, with the sampling weight for the i−th sample given by:


$$P(i)=\frac{I(i)}{\sum_{k=\text{1}}^{\left|B_{R}\right|}I(k)}$$ (14)

$B_{R}$ denotes the capacity of the prioritized replay Buffer. Trajectories not selected for the prioritized replay Buffer are stored in the uniform replay Buffer $B_{O}$. When $B_{O}$ exceeds its maximum capacity, old samples are discarded on a first-in-first-out basis. Samples in $B_{O}$ are assigned uniform sampling weights, meaning that each sample is selected with equal probability. The batch of samples used for gradient estimation is denoted as $B_{u}$, and is composed of samples from both $B_{R}$ and $B_{O}$:


$$B_{\mathrm{\backslash stixu}}=\zeta B_{R}+(\text{1}-\zeta )B_{O}$$ (15)

ζ∈[0,1] is a sampling ratio parameter used to balance the proportions of samples from the different replay Buffers.

An illustrative example of TPDEB:

Consider three policy replicas (workers) operating in parallel environments. Each worker collects a trajectory τ consisting of 10 transitions. For each trajectory, the cumulative TD-error ∆τ is computed. Suppose worker A collects a trajectory with ∆τ = 5.2, worker B with ∆τ = 2.1, and worker C with ∆τ = 7.8. Based on a predefined threshold, trajectories from worker A and C are stored in the prioritized buffer $B_{R}$, while the trajectory of B is stored in the uniform buffer $B_{O}$.

During training, the learner samples a batch Bu composed of 20% samples from $B_{R}$ and 80% from $B_{O}$ (ζ = 0.2). The importance weight of Each sample is calculated using Equation (13), and the policy gradient is updated accordingly. To stabilize learning across asynchronous replicas, a KL-divergence regularization term is added between the policy of learner and each worker.

This example demonstrates how TPDEB balances exploitation (high-TD-error samples) and exploration (recent uniform samples), while maintaining policy consistency via KL regularization.

#### 2.2.2. Dual experience replay buffer under the parallel framework.

**Fig 3:**
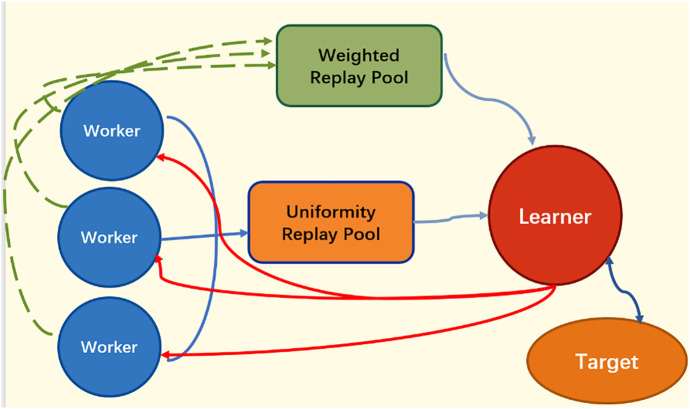
Dual experience replay buffer structure.

The dual experience replay Buffer is applied to the multi-policy replica architecture, as shown in Fig 3. Each worker continuously collects experience data in an independent thread and stores it in the weighted replay Buffer and the uniform replay Buffer, respectively. The learner samples batches of data from both replay Buffers for parameter updates according to Equation (15).

During training, the policy network uses the basic loss function defined by Equation (16):


$$L_{\pi }(\theta )=E_{s_{t},a_{t}}\left[R_{t}(\theta )\hat{A}(s_{t},a_{t})\right]$$ (16)

In addition, a regularization term is added to the loss function by incorporating the Kullback-Leibler (KL) divergence between $\pi_{\theta }$ and $\pi_{{worker}_{i}}$:


$$L_{D}(\theta )=\frac{\text{1}}{W}\sum_{i}KL\left(\pi_{\theta },\pi_{{worker}_{i}}\right)$$ (17)

The average KL divergence between the strategies of W workers and the learner is computed. Let the total objective function be:


$$L_{\Pi }(\theta )=L_{\pi }(\theta )+\eta L_{D}(\theta )$$ (18)

η is the regularization coefficient. The learner’s policy network is updated via gradient ascent:


$$\theta \leftarrow \theta +\varepsilon_{t}\nabla L_{\Pi }(\theta )=\theta +\varepsilon_{t}\left(\nabla_{\theta }L_{\pi }(\theta )+\eta L_{D}(\theta )\right)$$ (19)

Here, $\varepsilon_{t}$ denotes the learning rate. Policy gradient updates require the value function to estimate the TD error. The parameters ψ of the value function network are trained by minimizing the mean squared error of the TD error:


$$L_{v}(\psi )=E\left[{\left(v_{s_{t}}-V_{\psi }\right)}^{\text{2}}\right]$$ (20)

$$\nabla_{\psi }L_{v}(\psi )=\;E\left[-(v_{s_{t}}-V_{\psi })\nabla_{\psi }V_{\psi }(s_{t})\right]$$ (21)

$$\psi \leftarrow \psi -\xi_{t}\nabla_{\psi }L_{v}(\psi )$$ (22)

$\xi_{t}$ represents the learning rate for the value function, and $v_{s_{t}}$ is estimated using V-trace:


$$v_{s_{t}}=V_{\psi }\left(s_{t}\right)+\sum_{i=t}^{t+n-\text{1}}\gamma^{i-t}\left(\prod {\mathrm{\backslash nolimits}}_{j=t}^{i-\text{1}}c_{j}\right)Z_{i}$$ (23)

$Z_{i}=r_{i+\text{1}}+\gamma V_{\psi }\left(s_{i+\text{1}}\right)-V_{\psi }\left(s_{i}\right)$ denotes the temporal difference based on the state value function, with $\prod c_{i}=\text{1}$.

Algorithm 2 describes the process of the parallel policy gradient method based on the dual experience replay Buffer. This algorithm is abbreviated as TPDEB (Twin Prioritized Dual Experience Buffer) for ease of reference in subsequent experimental charts.

Algorithm 2. The Policy Gradient Algorithm Based on Dual Empirical Replay Buffer (TPDEB).

Inputs: number of samples used for one gradient estimation N, number of workers W, maximum capacity of weighted replay Buffer $M_{R}$, maximum capacity of uniform replay Buffer $M_{O}$, sampling ratio ζ, update step ε,ξ, number of training rounds $n_{episodes}$, update step of target policy network ς.

1: Initialisation:

2: Randomly initialise learner’s policy network θ and value function network ψ

3: Initialise the target strategy network $\theta^{\prime }\leftarrow \theta$

4: Initialise the policy network parameters of W workers as φ←θ

5: Initialise the dual experience replay Buffer using $M_{R},M_{O},\varphi$

6: Loop $j=\text{1},\cdots ,n_{episodes}$:

7: Sample a batch of interaction samples from the bi-experiential replay Buffer

$B_{u}=\zeta B_{R}+(\text{1}-\zeta )B_{O}$

8: Calculate the gradient of the policy network and the value function network

$R_{t}^{i}(\theta )=\frac{\nabla_{\theta }\pi_{\theta }(s_{t},a_{t})}{\text{max}\left(\pi_{target}\left(s_{t},a_{t}\right),\beta \pi_{{worker}_{i}}(s_{t},a_{t})\right)}$

$\begin{array}{c} \nabla_{\theta }L_{\Pi }(\theta )=\frac{\text{1}}{N}\sum_{i=\text{1}}^{N}R_{t}^{i}{\hat{A}}_{t}-\eta {\nabla_{\theta }L}_{D}(\theta ) \end{array}$

$\begin{array}{c} \nabla_{\psi }L_{v}(\psi )=\frac{\text{1}}{N}\sum_{i}^{N}\left(V_{\psi }\left(s_{i}\right)-v_{s_{i}}\right)\nabla_{\psi }V_{\psi }\left(s_{i}\right) \end{array}$

$\theta \leftarrow \theta +\varepsilon \nabla_{\theta }L_{\Pi }(\theta )$

$\psi \leftarrow \psi -\xi_{t}\nabla_{\psi }L_{v}(\psi )$

$\theta^{\prime }\leftarrow \varsigma \theta +(\text{1}-\varsigma )\theta^{\prime }$

9: until convergence or the maximum number of training rounds is reached.

**Fig 4:**
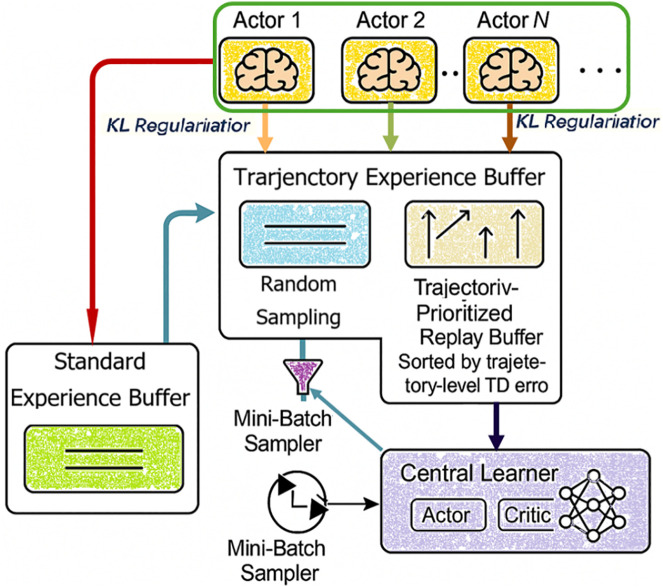
The overall framework of the TPDEB.

As illustrated in Fig 4 depicting the overall framework of TPDEB (Trajectory-Preserving Double Experience Buffer):

1) Preserve unmodified, uniform trajectories that reflect the true visitation distribution of the environment, which aids in stable value estimation.
2) Allow the prioritized buffer to focus exclusively on high-TD-error sequences, improving learning from rare but important events without introducing bias into the baseline distribution.
3) Integrates KL-regularization into actor updates to reduce policy divergence across replicas.

This architectural separation makes the learning pipeline more modular and better suited to our parallel policy setting, where each buffer can be sampled independently or adaptively prioritized.

## 3. Results

This paper evaluates the proposed method from two perspectives. First, ablation experiments are conducted to analyze the impact of the algorithm’s hyperparameters on performance. Next, cross-scenario comparative experiments are implemented to validate the advantages of the algorithm over existing methods.

### 3.1. Experimental environment

**Fig 5:**
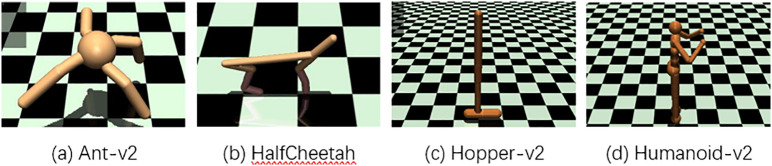
Partial sequential control task in OpenAI gym.

The experiments utilize robot mobility control tasks in MuJoCo [36] to investigate the impact of hyperparameters on performance. Fig 5 illustrates an example of the test tasks employed in the experiments. These tasks in Fig 5 are designed to enable robots with one or multiple legs to move forward quickly. As a 3D physics simulator, MuJoCo’s models fully take into account gravity and friction. The robot must achieve rapid movement by controlling the robot’s joints. A negative reward is assigned when the robot’s body contacts the ground. The dimensions of the state and action spaces for each task are presented in Table 1. Owing to their larger state and action spaces-which make it more challenging for the robot to master control strategies-the policy networks for Ant-v2 and Humanoid-v2 contain more hidden layer neurons than those for the other tasks.

**Table 1:** State and action space sizes of the robots in the experiment and the corresponding parameters of the algorithms used.

Robot name	State dimension	Motion Dimension	Neural Network Hidden Layer Shape	Discount factor ($\begin{array}{c} \gamma \end{array}$)
HalfCheetah-v2	17	6	64×64	0.99
Hopper-v2	11	3	64×64	0.99
Humanoid-v2	376	17	256×256	0.99
Ant-v2	111	8	256×256	0.99
Wal er2d-v2	17	6	64×64	0.99
S immer-v2	8	6	64×64	0.99

The experimental algorithm (Algorithm 2) is implemented using PyTorch, with deep neural network optimization performed via the Adam optimizer. The policy network and the value network share a similar structure: the input layer receives the observation vector, the hidden layer is composed of fully connected layers, and the output layer generates either the key parameters of the policy function or the estimated value function. Since the experimental environment is a robot motion control simulation with a continuous action space, the policy network outputs the Gaussian distribution parameters (mean and variance) for the actions. When executing actions, a specific action value is obtained by sampling from this distribution.

To account for differences in state dimensions across tasks, the scale of the network’s hidden layers is slightly adjusted; see Table 1 for the specific parameters. All experiments were conducted using 5 independent random seeds. The results presented in the figures reflect the mean performance across these five runs. and the training curves are plotted using a 20-step moving average. In each performance graph, the solid line represents the average value across all seeds, while the shaded area indicates the corresponding standard deviation. The shaded regions in each plot indicate ±1 standard deviation from the mean, calculated across the five random seed runs.

### 3.2. Ablation experiments

**Fig 6:**
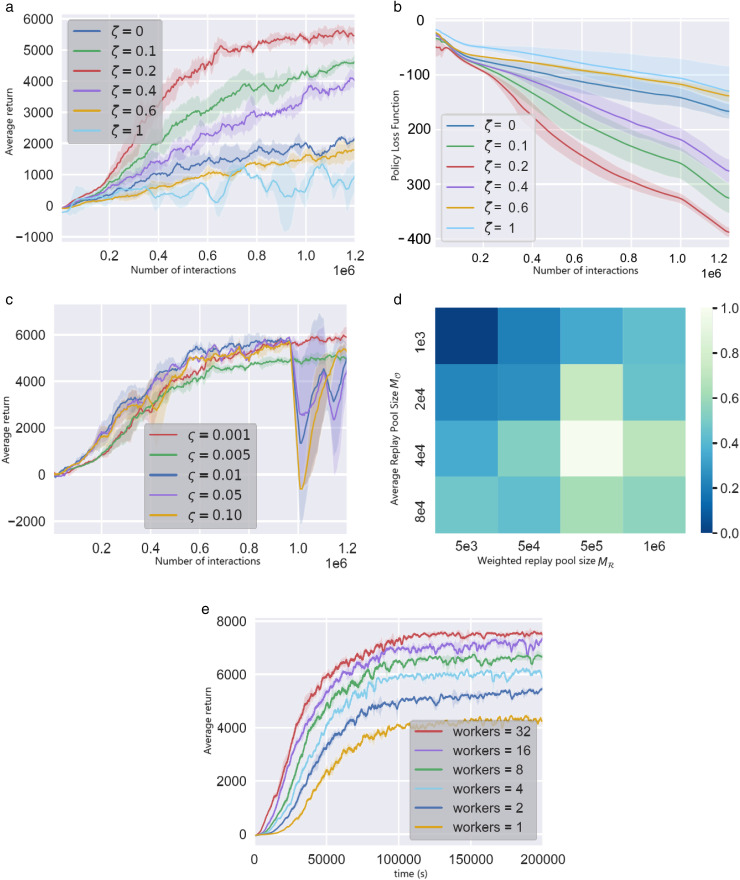
Results of Ablation Experiments in Ant-v2 Control Task. (a) Impact of dual-experience replenishment Buffer coefficients on returns. (b) Impact of dual empirical replay Buffer coefficients on strategy loss functions. (c) Impact of target strategy network update rate on performance. (d) Impact of bi-empirical replay Buffer size on performance. (e) Effect of number of workers on performance.

Ablation experiments are used to study the impact of the algorithm’s hyperparameters on performance. First, the effect of the dual experience replay Buffer coefficient ζ is analyzed: experiments on the Ant-v2 task were conducted with ζ set to 0, 0.1, 0.2, 0.4, 0.6, and 1, with results shown in Fig 6(a). As ζ increases, the proportion of $B_{R}$ in the gradient estimation samples rises, and performance exhibits an initial increase followed by a decline. This indicates the need to balance exploration and exploitation-$B_{R}$ samples reinforce the learning of critical experiences, while $B_{O}$ samples retain the most recent environmental information. When ζ=0, the algorithm degrades to a standard policy gradient method, yielding comparable performance. Experiments show that ζ=0.2 is the optimal choice for the Ant-v2 task. Fig 6(b) demonstrates the impact of ζ on the policy loss function: the loss decreases fastest when ζ=0.2. Together, these figures indicate that a proper setting of ζ can accelerate the convergence of the loss.

Another key parameter is the target policy network update rate, ς. Fig 6(c) reveals two phenomena: when ς is 0.01, 0.05, or 0.10, the robot’s performance drops sharply in the mid-training phase. Higher values of ς result in dramatic changes in the target network, which trigger fluctuations in gradient estimation and oscillations in trajectory returns. Conversely, when ς=0.01, the training remains stable but the performance is limited. This suggests that an excessively low value of ς can severely lag the target policy updates. The algorithm maintains a stable importance sampling factor by choosing an appropriate ς value.

Lastly, the effect of the experience replay Buffer capacity on the Ant-v2 task is investigated. $M_{R}$ is set to 5e3, 5e4, 5e5, and 1e6, while $M_{O}$ is set to 1e3, 2e4, 4e4, and 8e4 (with ζ=0.2). By varying $M_{R}$, the trajectory capacity K of the prioritized replay Buffer is implicitly controlled.

Fig 6(d) displays a heatmap of normalized returns for different capacity combinations: overall, increasing the replay Buffer size improves performance, but an excessively large prioritized Buffer can lead to performance degradation due to the difficulty of learning from outdated experiences.

Experimental tests evaluated the impact of varying numbers of workers on the robot’s performance, as shown in Fig 6(e). A greater number of workers can increase sample throughput, enabling the robot to achieve better performance in a shorter period. However, as the number of workers increases, the performance gains diminish. It should be noted that the algorithm’s computational complexity is related to the capacity of the weighted replay Buffer $B_{R}$. When a new trajectory is added to $B_{R}$, the sampling weights of all samples must be recalculated, resulting in an algorithmic complexity of $O(\left|B_{R}\right|)$.

### 3.3. Comparative experiments

In the comparative experiments, the main algorithms being compared are the high-performing IMPACT [37] and the distributed PPO [38] algorithm combined with PER. The IMPACT algorithm collects experience data in parallel using multiple policy replicas and employs a circular experience replay Buffer to enhance sample utilization. As a typical method of weighted replay, PER combined with distributed PPO is compared with the TPDEB algorithm presented in this chapter. Table 2 lists the six benchmark tasks involved in the experiments: HalfCheetah-v2, Hopper-v2, Humanoid-v2, Walker2d-v2, Ant-v2, and Swimmer-v2. Both the policy network and the value network utilize the tanh activation function, with the hidden layer structure detailed in Table 1. To ensure fairness, both PPO and IMPACT are implemented using the same network architecture. In the experiments, the prioritized replay Buffer coefficient is set to ζ = 0.2.

**Table 2:** Tasks, algorithms, and sample requirements in the comparison experiments.

Algorithms Samples Task	PPO	PPO with PER	IMPACT	TPDEB
Ant-v2	>1e6	>1e6	7.4e5	4.9e5
Humanoid-v2	>1e6	>1e6	8.4e5	3.9e5
HalfCheetah-v2	>1e6	>1e6	4.3e4	4.1e5
Hopper-v2	8.7e5	7.5e5	6.2e5	2.9e5
Walker2d-v2	>1e6	>1e6	4.6e5	4.2e5
Swimmer-v2	7.2e5	6.9e5	3.6e5	3.4e5

**Fig 7:**
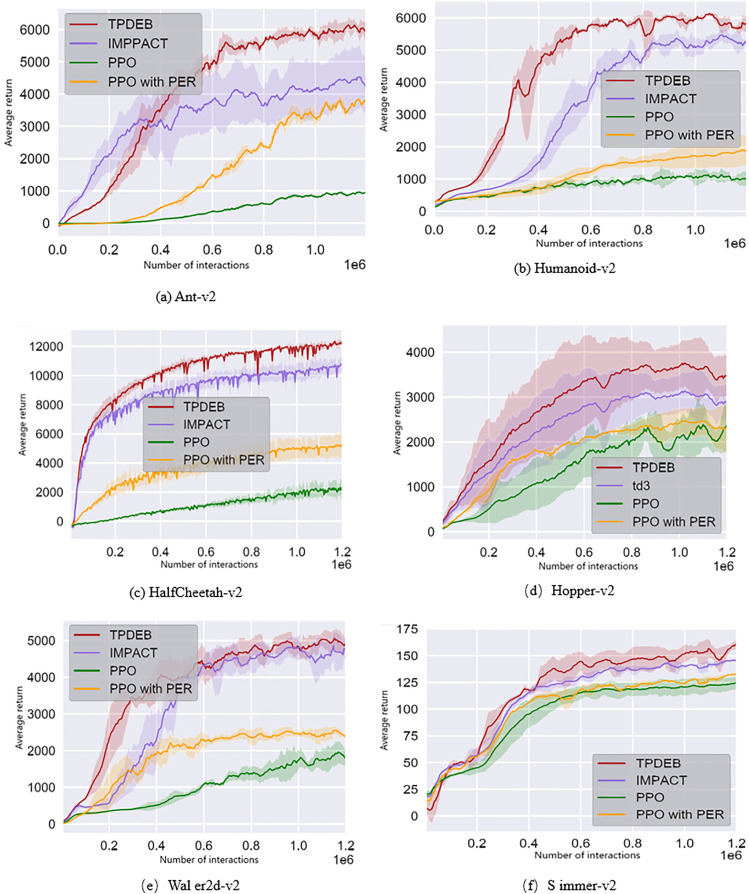
Results of comparison experiments with existing algorithms.

The learning curves of the agent in different tasks are shown in Fig 7. Overall, TPDEB exhibits significantly higher sample efficiency than IMPACT and PPO in most tasks. In particular, in challenging tasks such as Ant-v2 and Humanoid-v2, TPDEB achieves a higher average return. Table 2 compares the number of samples required by each algorithm to reach the same performance across different tasks, demonstrating that TPDEB shows a sample efficiency advantage in nearly all tasks, with its advantage becoming more pronounced as task difficulty increases.

## 4. Discussion

The theory and technology of single-agent reinforcement learning are the foundation supporting multi-agent reinforcement learning research. Reinforcement learning learns through a trial-and-error mechanism, often requiring a large amount of interaction data with the environment, which poses challenges for data collection and agent training. How to accelerate the acquisition of experience data, enabling agents to accumulate more interaction experience in a shorter time, is key to improving the efficiency of skill formation in agents. Using policy copies is an efficient parallel experience collection scheme, which involves copying multiple versions of the agent’s policy to collect experience data in parallel in their respective environments, significantly improving sample collection efficiency. During parallel training, the agent’s policy continuously updates and differs from the copies, requiring handling the impact of sample distribution differences on parameter updates. On the other hand, different experience data have varying learning values for agents, and existing uniform sampling methods do not consider the value differences between samples. It is necessary to distinguish the importance of interaction samples and improve sample utilization efficiency by assigning different sampling weights based on learning value. To address these challenges, this paper systematically analyzes the issue from the two perspectives of enhancing data collection speed and improving sample utilization efficiency, and proposes corresponding innovative solutions.

A high-efficiency parallel collection framework is proposed: deploying multi-threaded policy replicas to interact in independent environments in parallel to accelerate sample collection; correcting distribution shifts caused by policy differences via importance sampling; and introducing a target policy network along with a soft update mechanism to stabilize policy gradient estimation.

To improve the agent’s efficiency in learning from samples, a dual experience replay Buffer structure is designed. This structure comprises a weighted replay Buffer and a uniform replay Buffer. Samples in the weighted replay Buffer exhibit higher trajectory TD errors and are assigned different sampling weights based on their TD error. Samples in the uniform replay Buffer consist of those that could not be included in the weighted replay Buffer, and they are sampled uniformly. For each gradient estimation, the samples are drawn from both replay Buffers according to the sampling ratio ζ. By combining the parallel sample collection approach with the dual experience replay Buffer structure, a trajectory-based dual experience replay Buffer policy gradient algorithm is proposed.

In MuJoCo simulation experiments, the algorithm achieves optimal performance when the balance coefficient ζ is 0.2; comparative experiments show that the proposed algorithm significantly outperforms the baseline methods in both data collection speed and sample utilization efficiency.

Through the ablation experiments A greater number of workers can increase sample throughput, enabling the agent to achieve better performance in a shorter period. However, as the number of workers increases, the performance gains diminish. It should be noted that the algorithm’s computational complexity is related to the capacity of the weighted replay Buffer $B_{R}$. When a new trajectory is added to $B_{R}$, the sampling weights of all samples must be recalculated, resulting in an algorithmic complexity of $O(\left|B_{R}\right|)$.

Since TPDEB employs a weighted replay Buffer (PER) to enhance sample utilization, it is compared with algorithms that also utilize PER. In the experiments, the experience replay Buffer capacity for the PPO algorithm was expanded to three times its original size, with one-third of the samples retained for Buffer updates during each gradient update. Compared to PER-based PPO, TPDEB outperforms it in nearly all tasks. Its advantage stems from trajectory-level sampling probability calculations, which provide a more precise evaluation than PER’s single-sample approach, thereby accelerating the target policy learning process through efficient gradient updates.

Due to limitations in the author’s knowledge and capabilities, many aspects of the research require further in-depth exploration, detailed study, and thorough validation. Future research will mainly focus on:

The current validation is limited to simulation environments, and its adaptability to real-world application scenarios remains to be explored;

Further verification of the algorithm’s generalization across a diverse range of continuous action tasks is needed;

Promoting the practical deployment of the algorithm by extending it to multi agents path finding and dynamic obstacle avoidance, thereby addressing real-world deployment challenges.
